# Comparative Genomics of Cell Envelope Components in *Mycobacteria*

**DOI:** 10.1371/journal.pone.0019280

**Published:** 2011-05-06

**Authors:** Ruma Banerjee, Pankaj Vats, Sonal Dahale, Sunitha Manjari Kasibhatla, Rajendra Joshi

**Affiliations:** Bioinformatics Group, Centre for Development of Advanced Computing, Pune University Campus, Pune, Maharashtra, India; Institut de Pharmacologie et de Biologie Structurale, France

## Abstract

Mycobacterial cell envelope components have been a major focus of research due to their unique features that confer intrinsic resistance to antibiotics and chemicals apart from serving as a low-permeability barrier. The complex lipids secreted by *Mycobacteria* are known to evoke/repress host-immune response and thus contribute to its pathogenicity. This study focuses on the comparative genomics of the biosynthetic machinery of cell wall components across 21-mycobacterial genomes available in GenBank release 179.0. An insight into survival in varied environments could be attributed to its variation in the biosynthetic machinery. Gene-specific motifs like ‘DLLAQPTPAW’ of *ufaA1* gene, novel functional linkages such as involvement of Rv0227c in mycolate biosynthesis; Rv2613c in LAM biosynthesis and Rv1209 in arabinogalactan peptidoglycan biosynthesis were detected in this study. These predictions correlate well with the available mutant and coexpression data from TBDB. It also helped to arrive at a minimal functional gene set for these biosynthetic pathways that complements findings using TraSH.

## Introduction

The genus *Mycobacterium* includes bacteria known to cause dreadful diseases like tuberculosis, leprosy and skin ulcers [1]. One of the characteristic features of the members of this genus is the presence of a low-permeability cell envelope with high proportion of complex lipids that is organized into three superposed compartments viz., the plasma membrane, the cell wall skeleton and the capsule. Cell envelope components include mycolic acids, arabinogalactan (AG), lipoproteins, lipomannan (LM) and lipoarabinomannan (LAM). The pathogenicity and survival of *Mycobacterium* species in varied environments has been attributed to the variation in structural components of cell wall complex [2] and hence the variation in the biosynthetic machinery. The mycolic acid-arabinogalactan-peptidoglycan polymer is arranged to form a hydrophobic layer with other lipids [3], [4]. A variety of unique lipids, such as LAM, trehalose dimycolate and phthiocerol dimycocerate, anchor non-covalently with the cell membrane and appear to play an important role in virulence [5]. The enzymatic pathways that synthesize *M. tuberculosis* cell envelope lipids are the target of presently available antituberculosis antimicrobials and may be candidates for future antibiotic development.

In this study we have carried out comparative genomics of the biosynthetic machinery of cell wall components amongst 21-mycobacterial genomes using metabolic pathway context, sequence similarity tools and phylogenetic profiling. Phylogenetic profiling predicts functionally linked genes i.e., genes that are a part of a same biological process or cellular system based on the presence or absence of a protein in a set of reference genomes [6], [7]. Prediction of functional linkages helps in the annotation of uncharacterized genes thereby reducing the gap between rate of genome sequencing and annotation. Previous studies have established the fact that the conserved co-evolution patterns of gene-pairs across different genomes as suitable indicators of functionally linked genes [8], [9]. The predicted functional linked genes obtained by our studies were mapped with the co-expression data wherever available.

## Methods

The 21 mycobacterial genomes/proteomes included in the analysis were obtained from GenBank release 179.0 (Aug 15, 2010) [10] and are listed in Table 1.

**Table 1:** Details of the 21-mycobacterial genomes used for the comparative analysis.

Name	RefSeq ID/[Reference]	Abbreviation
*Mycobacterium abscessus ATCC 1997*	NC_010397/[11]	MAbATCC
*Mycobacterium avium 104*	NC_008595	MAv104
*Mycobacterium avium subsp. paratuberculosis K-10*	NC_002944/[12]	MAvK-10
*Mycobacterium bovis AF2122/97*	NC_002945/[13]	MBoAF
*Mycobacterium bovis BCG str. Pasteur 1173P2*	NC_008769/[14]	MBoBCG
*Mycobacterium bovis BCG str. Tokyo 172*	NC_012207/[15]	MBoTokyo
*Mycobacterium gilvum PYR-GCK*	NC_009338	MGlPYR
*Mycobacterium leprae Br4*	NC_011896/[16]	MLpBr
*Mycobacterium leprae TN*	NC_002677/[17]	MLpTN
*Mycobacterium marinum M*	NC_010612/[18]	MMrM
*Mycobacterium smegmatis str. MC2 155*	NC_008596	MSgMC2
*Mycobacterium sp. JLS*	NC_009077	MJLS
*Mycobacterium sp. KMS*	NC_008705	MKMS
*Mycobacterium sp. MCS*	NC_008146	MMCS
*Mycobacterium tuberculosis CDC1551*	NC_002755/[19]	MTbCDC
*Mycobacterium tuberculosis F11*	NC_009565	MTbF11
*Mycobacterium tuberculosis H37Ra*	NC_009525/[20]	MTbH37Ra
*Mycobacterium tuberculosis H37Rv*	NC_000962/[21]	MTbH37Rv
*Mycobacterium tuberculosis KZN 1435*	NC_012943	MTbKZN
*Mycobacterium ulcerans Agy99*	NC_008611/[22]	MUlAg
*Mycobacterium vanbaalenii PYR-1*	NC_008726	MVaPYR

Comparative genomics of the mycobacterial genomes with a focus on genes involved in biosynthesis of cell envelope components was carried out using methodologies *viz.,* sequence similarity, metabolic pathway reconstruction and phylogenetic profiling. Similarity search was carried out using MPI version of SSEARCH program available in FASTA3 package version 34 [23] with parameters E-value: e^−20^, %identity: 50 and %query coverage: 80. The query dataset used for the analysis is the proteome of *M. tuberculosis* H37Rv.

Multiple sequence alignment of proteins was carried out using parallel implementation of ClustalW-MPI version 0.13 [24]. Phylogenetic trees were reconstructed using parsimony (*protpars*) available in Phylip package version 3.67 [25]. Prior to phylogenetic reconstruction, to assess the statistical significance of the topology obtained, the data was bootstrapped to generate 100 data sets using *seqboot* program of Phylip. The trees were visualized using MEGA version 4 [26].

**Figure 1:**
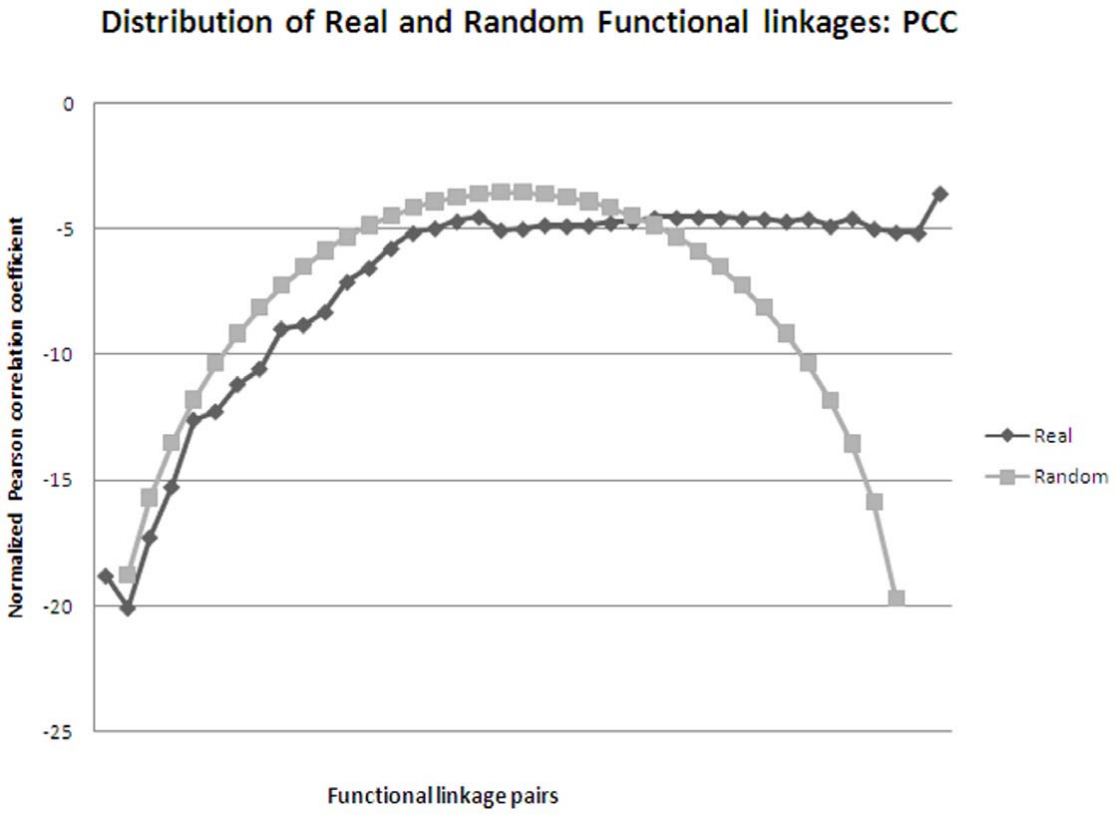
Distribution of Pearson correlation coefficient values for real and random datasets.

**Figure 2:**
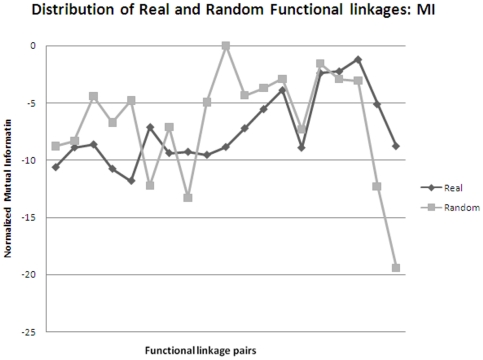
Plot of Mutual information values for real and random datasets.

Metabolic pathway reconstruction was carried out using Pathway Tools version 14.0 [27] with MetaCyc version 14.1 [28] as a reference database. As the curated genbank formatted file is the input for Pathway Tools, the genomes were curated with respect to functional information like ‘Enzyme Commission Number’ using the tool EFICAz2 version 13 [29] and UniProt database [30]. Metabolic pathways like LAM biosynthesis are unavailable in MetaCyc and were incorporated manually by mining of literature and using tools such as MarvinSketch [http://www.chemaxon.com/products/marvin/marvinsketch/] and ChemSpider [http://www.chemspider.com/] to add chemical structures of the metabolites involved. Pathway holes were filled using the ‘Power User’ mode with a probability cutoff of 0.9. Functionally linked genes were predicted using the methodology of phylogenetic profiling. The strength of this methodology lies in the fact that it takes into account the entire proteome and aids in the annotation of hypothetical genes that in-turn are capable of filling the pathway holes. Similarity profile matrix of all pairwise combination of genes was determined using SSEARCH, which implements the highly accurate and sensitive approach of dynamic programming for database similarity searching. The E-values obtained *via* SSEARCH were normalized [8] such that it enables capture of sequence divergence as well as generates continuous variables which are amenable to rigorous statistical treatment such as estimating mutual information content using B-spline function. Profile matrices were generated for both real as well as random datasets. Randomization aids in the calculation of ‘relationship-strength’ between gene-pairs that has occurred by chance and was implemented using *rand* function available in MATLAB [http://www.mathworks.com/]. Functionally linked genes were inferred based on calculation of mutual information content using *mis_calc* program [31], Pearson correlation coefficient and Hamming distance (using an *in-house* developed perl script). A plot of the above 3 parameters using both real and random data helped to generate the cutoff criteria for analyses of significant linkages (Figure 1 & Figure 2). Functionally linked genes in this study are defined as the genes that satisfy thecutoff criteria of 0.9 for mutual information content and 0.8 for Pearson correlation coefficient as these values differentiated the real from random data. Such stringent criteria were used as the comparison is between species belonging to the same genus viz., *Mycobacterium*. Hypothetical genes belonging to *MTBH37Rv* that exhibit conserved coevolution patterns in terms of similar profile to a well-characterized gene belonging to cell envelope biogenesis are analyzed in the present study. Genes displaying > = 50 functional linkages are predicted as ‘network-hubs’. The predicted functional linkages obtained were further substantiated by mapping withe co-expression data retrieved from TBDB [32] along with knockout mutants obtained *via* TraSH analysis [33]–[35] wherever available. For data management and efficient retrieval of ∼7700000 records, MySQL was used as DBMS. Anvaya, an *in-house* developed workflow environment that includes pre-defined workflows for ortholog identification, motif detection, phylogenetic reconstruction and phylogenetic profiling was used to perform all the above analysis.

## Results and Discussion

Comparative genomics of pathogenic and non-pathogenic *Mycobacteria* has played an instrumental role in unraveling many underlying factors responsible for virulence and host-specificity [36]. Detailed analysis of the biosynthetic machinery of mycolic acid, arabinogalactan, lipomannan/lipoarabinomannan, phthiocerol dimycocerate and lipoproteins is provided.

### Mycolate biosynthesis

Mycolic acids are alpha-alkyl, beta-hydroxy fatty acids and are the signature lipids of the hydrophobic mycobacterial cell wall [37]. The composition and quantities of mycolic acids are known to affect the virulence, growth rate, colony morphology and permeability of *Mycobacteria* [38]–[41]. Cells with reduced mycolate content show a higher permeability for substance uptake into the cells or excreted into the culture medium [42]. The biosynthesis pathway involves 5 different steps viz., production of malonyl CoA, fatty acid initiation (I, II and III) and fatty acid elongation (FAS I and FAS II) followed by the actual biosynthesis of mycolates. Altogether, 46 genes are known to participate in a coordinated manner to generate mycolates in *Mycobacteriaceae* members. Comparative analysis across the 21-mycobacterial genomes revealed that the biosynthetic pathway is conserved across all the species with differences arising due to redundant genes in different species.

### *fabH*

*fabH* gene is a pivotal link between Fatty acid biosynthesis I and II pathways [37]. It elongates acyl-CoA primers derived from FAS-I to form thioesters through condensation with malonyl-ACP. Current work revealed that *fabH* is absent in *M. leprae* strains and the domains are truncated either at C/N termini in *MMCS, MJLS, MKMS, MGlPYR and MSgMC2* and hence its functionality may be affected in these organisms. However, few studies report that the mycolate biosynthesis is not hindered, hence suggesting that there may exist alternate genes or pathways, which circumvent this step [43].

### *cmaA2, mmaA1 and umaA*

Cyclopropanation of mycolic acids is one of the distinguishing features of pathogenic *Mycobacteria* suggesting that this modification may be associated with an increase in oxidative stress experienced by the slow-growing species, and is catalysed by a family of S-adenosyl methionine dependent methyltransferases [44]. Even though the enzymes of this family share a conserved fold, they display high specificity for cis/trans cyclopropane ring formation in proximal/distal ends. These genes share a 50 to 70% identity between them; hence a lot of ambiguity arises in distinguishing these genes using sequence alignment tools alone. In order to detect sequence-motifs responsible for the observed specificity, we retrieved all the methyl transferase genes (totaling 170) from the 21 *Mycobacteria* and subjected them to multiple sequence alignment (MSA).

**Figure 3:**
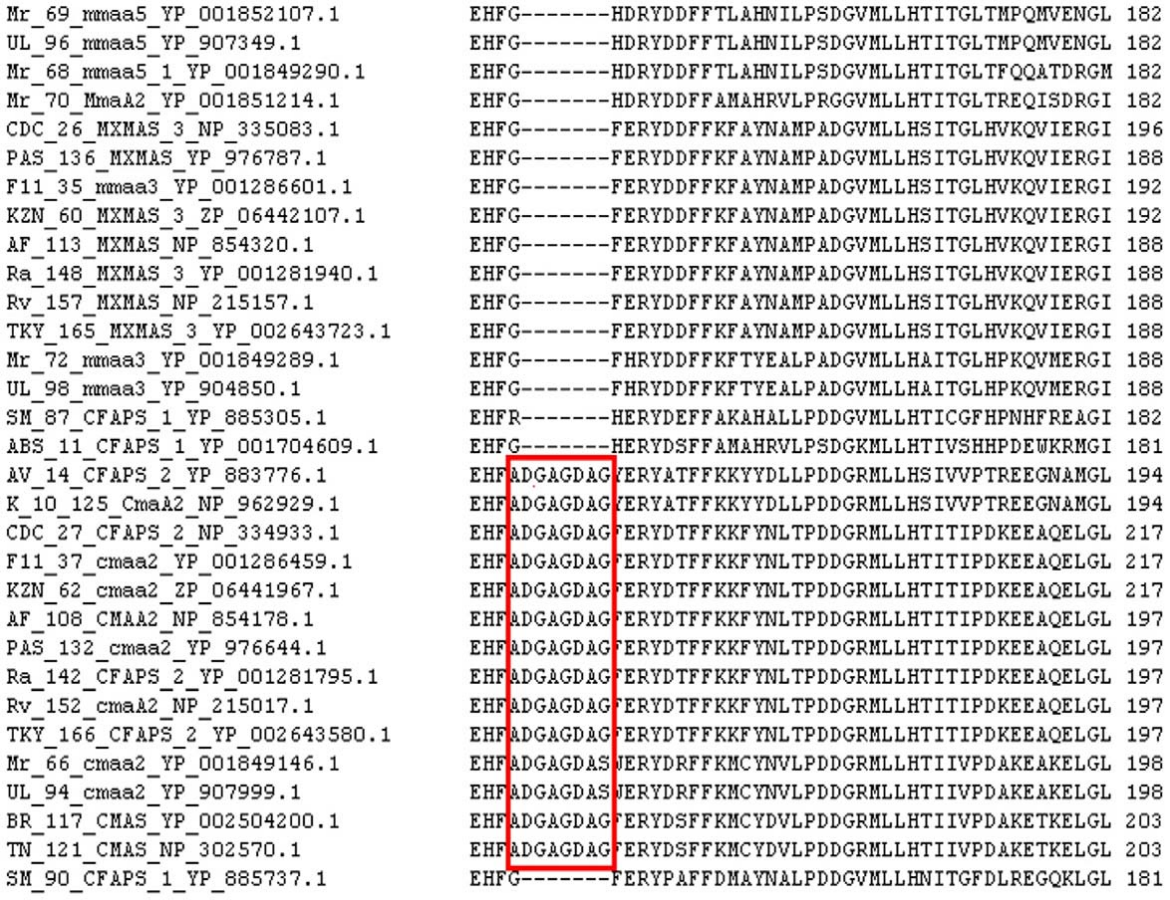
MSA of cyclopropane synthases depicting the ‘ADGADAG’ motif in cma2 gene.

The MSA (Figure 3) clearly depicted that ‘ADGAGDA’ motif is unique to *cmaA2* (RefSeq ID: NP_215017), which encodes the enzyme responsible for trans-cyclopropanation at the proximal end of meromycolate chain as reported earlier [45]. This hydrophilic stretch is surface accessible inspite of being adjacent to binding site residues (which are buried in the hydrophobic core) and forms a loop away from the active site. Recent reports suggest that *cmaA2* carries out both cis and trans cyclopropanation at the proximal position of the oxygenated mycolates [46].

A protein of *MLpTN* with NCBI accession id NP_302570.1 is annotated as a cyclopropane synthase. This protein may be re-annotated as *cmaA2* as it contains the *cmaA2*-specific ‘ADGAGDA’ motif. The study also revealed that *cmaA2* gene is absent in fast growing species, *MAbATCC, MGlPYR, MVaPYR, MSgMC2, MJLS, MKMS and MMCS* as the corresponding orthologues do not contain the ‘ADGAGDA’ motif (Figure 3). Hence in the above listed organisms, trans cyclopropanation at proximal end may not take place, as this reaction is specific to *cmaA2.* This further reiterates the rationale for the implication of *cmaA2* as one of the major factors contributing to the pathogenicity of *Mycobacteria* as mutants lacking this gene are known to evoke 5-fold increase in host – immune response [47].

**Figure 4:**
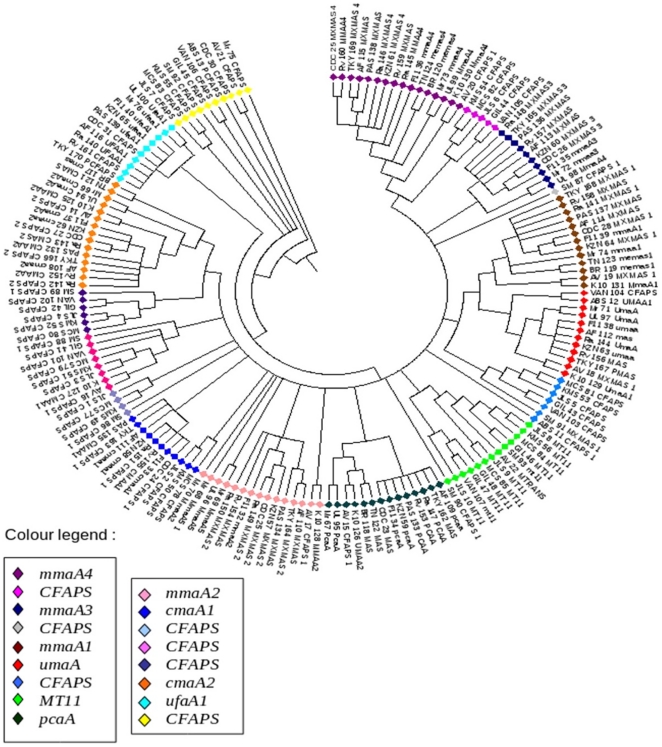
Phylogenetic tree of 170 cyclopropane synthases from Mycobacteria.

In order to gain further insight into the relationship amongst the several cyclopropane synthases, a phylogenetic tree was reconstructed using parsimony (Figure 4). An in-depth analysis of the phylogenetic tree revealed that *mmaA1* responsible for methylation at the proximal end of mycolic acid [44] is present in *MTbH37Rv, MBoAF, MLpTN and MAv104* strains and forms a distinct cluster. Methylation and cis-trans isomerization by *mmaA1* is succeeded by trans cyclopropanation by *cmaA2*. It is interesting to observe that all organisms containing *cmaA2* also contain *mmaA1* except *MUlAg*. However, it needs to be mentioned here that the *mmaA1* cluster is shared with another node that contain *umaA* genes with significant bootstrap value (97 times out of 100) and *MUlAg* contains one such *umaA* gene (Locus tag: MUL_4538). The *mmaA1* cluster also includes a node with a small cluster of genes without any specific functionality but contain the cyclopropane synthase domain. These genes belong to *MSgMC2, MJLS, MKMS, MMCS, MGlPYR, MVaPYR and MAbATCC.*

### mmaA2

*mmaA2* has two distinct roles viz., cis cyclopropanation at distal end in alpha mycolates and cis cyclopropanation at proximal end in oxygenated mycolates [48]. Previous experiments have shown that distal cyclopropanation helps in increasing the resistance of *Mycobacteria* to hydrogen peroxide, a major factor contributing to the oxidative stress experienced by the bacteria [49]. *mmaA2* is absent in non-pathogenic strains such as, *MJLS, MKMS, MMCS, MGlPYR, MSgMC2* and *MVaPYR*. In the phylogenetic tree, these genes form a distinct cluster.

### mmaA3

*mmaA3* catalyses the addition of methyl moiety at the hydroxyl group which is newly formed by *mmaA4* at the distal end in oxygenated mycolates [50], [37], [38]. It is interesting to note that *mmaA3* is absent in *MLpTN, MLpBr*, *MAvK-10* and *MAv104*, hence may affect the methoxy mycolate production in these organisms. In the phylogenetic tree *mmaA3* cluster share the same node with *mmaA4* cluster.

### mmaA4

*mmaA4* catalyses the addition of methyl and hydroxyl branch at distal end in oxygenated mycolates [51], [52]. Deletion of *mmaA4* abolishes synthesis of both methoxy- and ketomycolates in *Mycobacteria* [24], [31]. The node containing *mmaA4* also includes a set of genes which do not have well defined function but contain the methyltransferase domain. These ‘undefined methyltransferase genes’ belong to non-pathogenic strains such as *MJLS, MKMS, MMCS, MGlPY* and *MVaPYR.* It is interesting to note that these organisms do not contain *mmaA4*. Hence it can hypothesized that these undefined methyltransferase genes may have function similar to *mmaA4* as absence of *mmaA4* leads to reduction in ketomycolate production, causing increased permeability of the cell wall, with the hypersensitivity to both ampicillin and RIF and impaired growth at low temperature [41].

### ufaA1

**Figure 5:**
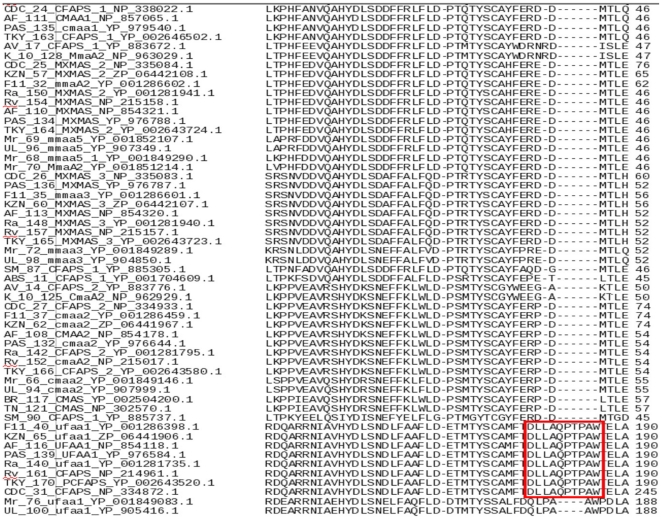
MSA of cylcopropane synthases depicting the ‘DLLAQPTPAW’ motif in ufaA1 gene.

*ufaA1* and other cyclopropane synthase containing proteins cluster separately. MSA of *ufaA1* genes helped to delineate the motif ‘DLLAQPTPAW’ (Figure 5). The orthologues of this gene are absent in *MAbATCC*, *MVaPYR*, *MSgMC2, MJLS, MKMS, MMCS, MGlPYR, MLpTN, MLpBr, MAvK-10* and *MAv104* as the motif ‘DLLAQPTPAW’ is absent. However, the affect of the absence of ufaA1 in mycolate biosynthesis needs to be probed further. This motif is however not conserved in *ufaA1* orthologs of *MMrM* and *MUlAg*. This observation can be attributed to the fact that both these organisms have taxonomically distinct relationship in comparison to other *mycobacteria* and are known to produce stereochemically different mycolates [53].

### pcaA

The cluster of *pcaA* that carries out the proximal cyclopropanation of alpha-mycolates [39] is shared with MT-11. *pcaA* is uniquely present only in pathogenic strains viz., *MTbH37Rv, MTbH37Ra, MtbCDC, MBoAF MboTokyo, MBoBCG, MKZN, MTbF11, MAv104, MAvK-10, MMrM, MulAg, MLpTN* and *MLpBr.* The phylogenetic tree depicts that *MAv104* (Locus tag: MAV_4679) and *MTbCDC* (Locus tag: MT0486) are *pcaA* genes even though the same is not annotated accordingly in public domain databases.

### MT-11

Methyltransferase type-11 (MT-11) is involved in DNA regulation. These genes have been picked up during the search for ‘methyltransferase containing domains’. They however do not have a direct evident role in mycolate biosynthesis and hence were not analysed further. It is however interesting to note that MT-11 is present only in *MVaPYR*, *MSgMC2, MJLS, MKMS, MMCS, MGlPYR, MMrM, MAvK-10* and *MAv104.*

### otsB2

*otsB2* encodes trehalose-6-phosphate phosphatase which dephosphorylates trehalose-6-phosphate to yield trehalose-mono-phosphate. The truncation of *otsB2* at N' in *MSgMC2* was observed during the comparative analysis, and may have affect on its functionality. However, studies by Woodruff et al., [54] revealed that this gene is functional.

### Predicted functional linkages

**Figure 6:**
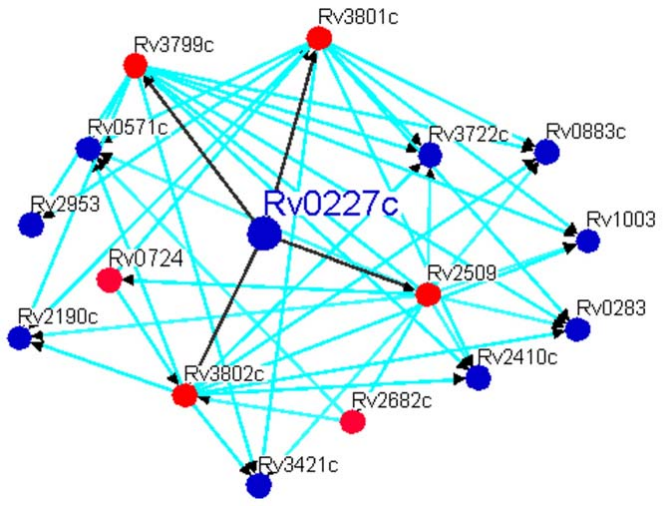
Predicted functional linkages of Rv0227c. Nodes in red colour are genes with known function in mycolate biosynthesis while those in blue colour are hypothetical genes. Black line shows direct linkage of Rv0227c.

Novel functional linkages could be identified for genes involved in mycolate biosynthesis using phylogenetic profiling. These linkages are said to be novel, as the present annotation does not suggest any definite role of these genes in mycolic acid biosynthesis. A total of 1661 unique protein pairs satisfied the criteria for MI and CC (Table S1). Of these, 91 proteins have well-defined function in public domain databases like Tuberculist [http://tuberculist.epfl.ch/] and TBDB. Analysis of the proteins with known function revealed that Rv0503c (*cmaA2* involved in trans cyclopropanation at proximal end of mycolate), Rv0470c (*pcaA* involved in cis cyclopropanation at proximal end of mycolate), Rv1273c (ABC transporter) and Rv3804c (*fbpA* involved in cell wall biosynthesis via its mycolyltransferase activity) display >230 functional linkages (Table 2). These genes can thus serve as network hubs [55] and may be probable chokepoints. This hypothesis is further substantiated by the crucial role played by these genes in mycolate biosynthesis as deduced by several experimental studies pertaining to knockout mutants[33]–[35], [39], [56]–[58] and co-expression[TBDB] [33]–[35] For e.g., Rv0227c (a conserved hypothetical protein) shares significant MI and PCC with mycolate genes like Rv3801c (*fadD32*) and Rv3799c (*accD4*), and hence, Rv0227c may be functionally linked to mycolic acid biosynthesis (Figure 6). It has also been proved to be essential for growth by Sassetti et al, 2003 [33]–[35] and co-expression values with *fadD32* and *accD4* is 0.43 and 0.61 respectively. Hence, it can be hypothesized that this gene seems to have a vital role in mycolate synthesis.

**Table 2:** Genes with >50 functional linkages across the biosynthentic pathways of cell wall components.

Pathways	Genes	Number of functional linkages
**Mycolate biosynthesis**	Rv3804c	242
	Rv1273c	241
	Rv0503c	240
	Rv0470c	239
	Rv3280	99
	Rv3802c	95
	Rv3801c	94
	Rv3799c	93
	Rv2509	92
	Rv1484	65
	Rv0644c	51
	Rv0643c	50
**Arabinogalactan biosynthesis**	Rv3809c	248
	Rv3794	246
	Rv3464	243
	Rv3265c	242
	Rv2361c	240
	Rv1086	237
	Rv1302	236
	Rv2152c	113
	Rv1315	111
	Rv2682c	95
	Rv2155c	65
**LAM biosynthesis**	Rv2174	242
	Rv2188c	112
	Rv2610c	112
**PDIM biosynthesis**	Rv1528c	99
	Rv2941	98
	Rv3820c	98
	Rv2930	96
	Rv3824c	95
	Rv2942	93
	Rv2933	91

The hypothetical proteins which share significant MI (>0.9) and PCC (>0.8) with genes involved in mycolate biosynthesis, and their role is further substantiated by recent literature, were summarized in Table 3. When the functional linkages displayed by the hypothetical proteins predicted to be involved in mycolate biosynthesis were analysed (Table 3), the following were the observations. Rv3802c recently annotated as phospholipase thioesterase [58], displayed maximum linkages (total: 121). Based on these observations it can be hence hypothesized that this enzyme may play a crucial role in mycolate biosynthesis and hints at the possibility that the functionality encoded by this gene is unique. Rv3722c a conserved hypothetical gene, shown to be essential by Sasetti et al, 2003[34], [35], displays significant co-expression with mycolate biosynthesis genes, hence providing a possible role of this gene in this pathway.

**Table 3:** Hypothetical proteins predicted to be functionally linked with mycolate biosynthesis.

Hypothetical protein	Mycolate pathway genes	MI	co-expression	PCC	Predicted domain in hypothetical protein	Mutant studies	Supporting literature
Rv2141c	Rv0636	0.992774	0.41302	0.872206	Peptidase family M20/M25/M40	Non-essential	Probable dapE2 Soluble protein [90]
Rv1784	Rv0503c	0.934068	0.45765	0.909174	FtsK/SpoIIIE family	no data available	Rv1784 is a split gene; supposed to be a complete gene by adding 1784 and 1783 Rv numbers.[92]
Rv3722c	Rv3799c	0.992774	0.50171	0.971905	N/A	Essential	co-transcribed with sRNA [87]
Rv3031	Rv3804c	0.934068	0.50882	0.951319	Glycosyl hydrolase family 57/Domain of unknown function (DUF1957)	Essential	(Rv3031) likely to be involved in the formation of the alpha-(1R6)-glycosidic bond [59]
Rv3908	Rv0636	0.992774	0.5198	0.929026	NUDIX domain	Non-essential	nudix ptotein shows a role as antimutator in M.smegmatis [88]
Rv2953	Rv3799c	0.992774	0.53548	0.965918	Saccharopine dehydrogenase	Non-essential	Putatively encodes an enoyl reductase. [89], [93]
Rv3802c	Rv0957	0.992774	−0.33797	0.979063	cutinase	Essential	Rv3802c is involved in joining the mero and a mycolate into a mature mycolic acid [58]
Rv3802c	Rv0724	0.992774	0.57179	0.992004	cutinase	Essential	Rv3802c is involved in joining the mero and a mycolate into a mature mycolic acid and transferring it to trehalose or arabinogalactan are located in a gene cluster from Rv3799c to at least Rv3804c [58]

Please refer to the Table S1 for the complete list of hypothetical proteins that share significant functional linkages with mycolate biosynthesis genes, and hence may play a role either in transport of the protein products or in the regulation of the genes.

### Arabinogalactan biosynthesis

Arabinogalactan tethers the mycolic acid layer to the peptidoglycan [59], [60]. The biosynthesis of AG has been reviewed extensively and the same is included as a component of ‘mycolyl-arabinogalactan-peptidoglycan complex’ biosynthesis in MetaCyc. Comparative analyses revealed that all the genes involved in AG biosynthesis are conserved in the 21-mycobacterial genomes with minor differences arising in genes with redundant functionality. *rmlC* gene encodes dTDP-4-keto-6-deoxyglucose epimerase, the third enzyme in the *M. tuberculosis* dTDP-L-rhamnose pathway. *rmlC* is conserved in all organisms except *MGlPYR* wherein it is present as a bifunctional protein (Locus tag: Mflv_3297) possessing truncated reductase and epimerase domains. Rv3779 is a glycosyltransferase responsible for direct synthesis of polyprenyl-phospho-mannopyranose, an intermediate in AG biosynthesis [61]. The orthologs of this gene are absent in fast-growing species like *MGlPYR, MVaPYR*, *MSgMC2, MJLS, MKMS* and *MMCS* and hence may be responsible for the observed variations in cell growth and shape in these organisms.

### Phylogenetic profiling

The number of predicted functionally linked protein pairs for AG biosynthesis is 2086. These include, Rv1302 (*rfe*, involved in AG biosynthesis), Rv1086 (short-chain isoprenyl diphosphate synthase), Rv2361c (long-chain isoprenyl diphosphate synthase; essential gene), Rv3265c (*wbbL1*, rhamnosyl transferase; essential gene), Rv3464 (*rmlB*, dTDP-GLUCOSE 4,6-DEHYDRATASE; essential gene), Rv3794 (*embA*, arabinosyl transferase; essential gene) and Rv3809c (*glf*, galactose pyrannose mutase) and display >230 functional linkages (Table 2). Mutant studies for the above proteins provide evidence for them to be “essential” for survival [33]–[35]. The large number of functional linkages displayed by these proteins when correlated with mutant studies suggest their role as ‘hubs’ in protein interaction networks, which may further translate into them being “choke points” in metabolic networks. Thus, the high proportion of functional linkages correlate directly with the mutant studies thus providing a rationale for the hypothesis that number of linkages is directly proportional to the essentiality of the gene.

The hypothetical proteins which share significant MI (>0.9) and PCC (>0.9) with genes involved in AG biosynthesis, and their role is further substantiated by recent literature, were summarized in Table 4. Genes Rv1209c and Rv3031 are annotated as ‘hypothetical proteins’ and share significant mutual information content and Pearson correlation coefficient with genes involved in AG biosynthesis, hence suggesting a role of these genes in AG metabolism (Table 4). Recent findings by Jackson et al., 2009 [62] and Kaur et al., 2009 [59] support the same. Please refer to the Table S2 for the complete list of hypothetical proteins that share significant functional linkages with AG biosynthesis genes.

**Table 4:** Hypothetical proteins predicted to be functionally linked with AG biosynthesis.

Hypothetical protein	AG biosynthesis genes	MI	Co-Exp	PCC	Predicted Domain in hypothetical protein	Mutant studies	Supporting Literature
Rv1209	Rv3265c	0.934068	0.47394	0.965525	N/A	Non-essential	probably involved in cell wall arabinogalactan linker formation uses DTP rhamnosyl residue into cell wall[59], [62]
Rv3031	Rv3265c	0.934068	0.39593	0.962174	Glycosyl hydrolase family 57/Domain of unknown function (DUF1957)	Essential	(Rv3031) likely to be involved in the formation of the alpha-(1R6)-glycosidic bond linking the first and second D-Glcp residues at the reducing end of the molecule.[59]

### Lipomannan and lipoarabinomannan biosynthesis

Lipoarabinomannan and lipomannan phosphatidylinositol mannosides (PIMs) are major phosphatidylinositol (PI)-based lipoglycans/glycolipids of *Mycobacterium*. They play a major role in phagocytosis, persistence of bacilli in phagocytic cells, CD-1-restricted antigen presentation, initiation of innate immunity, and in antibody-mediated immunity [2]. Recent work of Kaur D et al., 2009 [59] has provided a thorough understanding of LM and LAM biosynthesis. However the same is not included in public-domain databases.

For comparative analysis, an exclusive list of genes catalyzing the LM/LAM biosynthesis process is mandatory. In order to facilitate ease-of-mapping across 21 genomes, we have reconstructed the pathway manually via literature curation and Pathway Tools software. Some of the interesting observations apart from the variations reported earlier in Kaur D et al., 2009 [59] in genes responsible for capping includes Rv2181, an integral membrane protein. It is the alpha (1→2) ManT responsible for the synthesis of the alpha (1→2) ManP-linked branches, characteristic of the mannan backbone of LM and LAM; orthologous gene of which is absent in *MAbATCC* as it may contain unusual alpha (1→3) mannosyl side chains as in *M. chelonae*, [63] instead of alpha (1→2) which is commonly found in all other mycobacterial species [59], [64], [65]. Rv1635c is a transmembrane protein that carries out mannose capping of LAM moieties in the periplasmic side of plasmamembrane in *MTbH37Rv* [59]. The orthologs of this gene are absent in *MSgMC2, MKMS, MMCS, MJLS* and *MAbATCC,* as these species are devoid of ManLAM and contain alternate capping of LAM moieties viz., PILAM and AraLAM [66]–[71], [59].

473 genes were predicted to be functionally linked (via phylogenetic profiling) with genes known to be involved in LAM biosynthesis (Table 2). Rv2174 (mptA), a polyprenol-P- mannose alpha 1→6 mannosyltransferase displayed the maximum linkages (>230). The large number of linkages can be attributed to the fact that disruption of this gene affects the optimal growth of the mycobacterium [34], [35] as revealed by the TraSH analysis of this gene. Rv2188c, *pimB,* an alpha-D-Mannose-alpha 1→6-phosphatidyl-myoinositol mannosyltransferase and Rv2610c (*pimA*) display 112 linkages each, thus providing a rationale for their inclusion as potential candidates for therapeutic development.

The hypothetical proteins which share significant MI (>0.9) and PCC (>0.9) with genes involved in LAM biosynthesis, and their role is further substantiated by recent literature, were summarized in Table 5. Few studies have found evidence that Rv2613c encodes GT4 family glycosyl transferase [72] and Rv2257c encodes a homologue of *pbpX* of *MSgMC2*, which has a role in antibiotic resistance [73]. Table S3 catalogs the complete list of hypothetical genes that may have a possible role in LAM biosynthesis.

**Table 5:** Hypothetical genes predicted to be functionally linked with LAM biosynthesis.

Hypothetical protein	LAM biosynthesis genes	MI	Co-Exp	PCC	Predicted Domain in hypothetical protein	Mutant studies	Supporting Literature
Rv2613c	Rv2610c	0.992774	0.5078	0.981656	HIT domain	Essential	Belongs to the large GT4 family of glycosyl transferases, [72]
Rv0263c	Rv2610c	0.992774	−0.46628	0.972042	Allophanate hydrolase subunit 2	Non-essential	putative carboxylase catalyzing urea degradation [91]
Rv2257c	Rv2611c	1	0.47046	0.982687	Beta-lactamase	Non-essential	homologue of pbpX (M.smegmatis) which play a role in encoding beta-lactam antibiotic-resistant enzymes [73]

### Phthiocerol dimycocerosate (PDIM) biosynthesis

PDIM has a prominent role in evoking adaptive immune response as well as in combating oxidative stress by scavenging the oxygen free radicals [74]. PDIM biosynthesis involves four main steps viz., priming of long-fatty acids and synthesis of diol component of phthiocerol, biosynthesis of phthiocerol *ppsE* protein, enzymatic synthesis of mycocerosic acid and transesterification of mycocerosic acid onto the diol component of phthiocerol [75]. It is now understood that the complete PDIM molecules are synthesized in the cytoplasm of *M. tuberculosis* before being translocated into the cell wall [76]. Previous studies have reported the presence of PDIM in pathogenic *Mycobacteria* with the exception of *M.gastri* [74]. PDIM biosynthetic pathway has been reconstructed *in-house* using Pathway Tools. The biosynthesis of PDIM is carried out by genes *ppsA-E,* which encode a type I modular polyketide synthase responsible for the synthesis of phthiocerol and phenolphthiocerol by elongation of a C20–C22 fatty acyl chain or an acyl chain containing a phenol moiety with three malonyl-CoA and two methylmalonyl-CoA units [77]. *mas* encodes an iterative type I polyketide synthase that produces mycocerosic acids after two to four rounds of extension of C18–C20 fatty acids with methylmalonyl-CoA units [78]. *papA5* catalyzes diesterification of phthiocerol and phthiodiolone with mycocerosate [79] along with *fadD26*, a fatty acyl- AMP ligase involved in the activation and transfer of long-chain fatty acids [80]. *drrC* and *mmpL7* are necessary for the proper localization of DIM [76].

Comparative studies revealed that FadD26 is present in all 21 mycobacterial species. *ppsA-E* genes are absent in *MAv104, MAvK-10, MSgMC2, MAbATCC, MKMS*, *MJLS* and *MMCS*. *ppsC-E* genes are absent in *MVaPYR* and *papA5* is absent in *MAv104, MAvK-10* and *MSgMC2.* The gene *mas* is absent in *MVaPYR* and *MGlPYR.* These findings hints at the absence of PDIM production in the organisms listed above. The transport proteins of PDIM viz., *mmpl7* is absent in *MAv104, MAvK-10, MSgMC2, MKMS*, *MJLS*, *MMCS*, *MGlPYR* and *MVaPYR; drrC* is however present in all the 21 *Mycobacteria*. This suggests that the daunorubicin resistance, which is one of the activities of *drrC* apart from its role in PDIM translocation, is present is these organisms inspite the absence PDIM production.

### Phylogenetic profiling for PDIM biosynthesis

A total of 677 unique protein pairs satisfied the criteria for MI and CC and were predicted to be functionally linked (via phylogenetic profiling) with genes known to be involved in PDIM biosynthesis (Table S4). Rv1528c (polyketide synthase associated protein *PapA4*) displayed the maximum number of linkages (99). Analysis of the proteins with known function revealed that Rv2933 (*rfe*, involved in PDIM biosynthesis), Rv2930 (acyl-CoA synthetase), Rv2941 (acyl-CoA synthetase), Rv2942 (transmembrane transport protein *MmpL7*), Rv3820c (polyketide synthase associated protein *PapA2*) and Rv3824c (polyketide synthase associated protein *PapA1*) displayed >90 functional linkages (Table 2).

The hypothetical proteins which share significant MI (>0.9) and PCC (>0.8) with genes involved in PDIM biosynthesis, and their role is further substantiated by literature, are summarized in Table 6. Rv0748, Rv1301 and Rv2681, annotated as ‘conserved hypothetical proteins’ may have a role to play in in this pathway as they share significant MI and PCC with PDIM genes and have also been shown to be essential by mutant studies. Rv1461, a conserved hypothetical protein, shown to be essential by Sasseti et al., 2003, displays significant co-expression with Rv2930 (acyl- CoA synthetase). Previous studies report that Rv1461 (*ppS1*) to be an ortholog of *SufB*, a highly conserved component of the [Fe-S] cluster, assembly and repair SUF (mobilization of sulfur) machinery, crucial for survival [81]. This clearly shows the crucial role of this enzyme in PDIM biosynthesis and hints at the possibility that the functionality encoded by this gene is unique. Rv2681, a conserved hypothetical protein, shows significant co-expression with Rv2933 and Rv2942, hence providing a possible role of this gene in this pathway [82]. Please refer to the Table S4 for the complete list of hypothetical proteins that share significant functional linkages with PDIM biosynthesis genes.

**Table 6:** Hypothetical genes predicted to be functionally linked with PDIM biosynthesis.

Hypothetical protein	PDIM Biosynthesis	Mutual information	Co-Exp	PCC	Predicted Domain in hypothetical protein	Mutant study	Function of gene involved in PDIM biosynthesis
Rv2681	Rv2933	0.992774	−0.30528	0.92159	3′-5′ exonuclease/HRDC domain	slow growth mutant	phenolpthiocerol synthesis type-I polyketide
Rv1461	Rv2930	0.934068	0.33547	0.968496	Hom_end-associated Hint/Uncharacterized protein family (UPF0051)	essential	acyl-CoA synthetase
Rv2681	Rv2942	0.992774	0.38414	0.923044	3′-5′ exonuclease/HRDC domain	slow growth mutant	transmembrane transport protein MmpL7
Rv1301	Rv2933	0.992774	0.47968	0.876667	yrdC domain	essential	phenolpthiocerol synthesis type-I polyketide
Rv0748	Rv3824c	0.934068	0.48618	0.910001	Ribbon-helix-helix protein, copG family	non essential	polyketide synthase associated protein

### Identification of putative drug targets

The worldwide increase in multi-drug resistant *Mycobacterium tuberculosis* strains poses a great threat to human health and highlights the need to identify new anti-tubercular agents. The construction and analysis of molecular interaction networks provides a powerful means to understand the complexity of biological systems and to reveal hidden relationships between drugs, genes, proteins, and diseases [83]. We have used the knowledge gained from the above analyses for rational identification of putative drug targets and estimated their appropriateness by sequence analysis. Many currently unexploited *MTbH37Rv* receptors may be chemically druggable and could serve as novel anti-tubercular targets. Those genes in the above analysis that were classified as essential, automatically form a first list of putative targets for anti-tubercular drugs, since their total inactivation may result in loss of production of cell wall components and hence the viability or the pathogenicity of the bacteria. However, it is reasoned that an ideal target should be essential not only in terms of the reaction it can catalyse, but also as the only protein coded by the genome that can perform the same task. Moreover, an ideal target should also have no recognisable homologue in the host system, which can in principle compete with the same drug, leading to adverse effects in the host system [84], [85]. Sequence analysis of *MTbH37Rv* with human proteomes was therefore carried out for each of the identified targets and the results are summarised in Table 7.

**Table 7:** Functionally linked genes with cell wall components and their homolog information in humans.

Functionally linked genes with cell wall components	Paralogs of *MtbH37Rv*				Homologs in Human			
Rv_number	Hit found	E-value <0.0001	Identity <40(%)	Query coverage <30(%)	Hit found	E-value <0.0001	Identity <40(%)	Query coverage <30(%)
Rv3802c	No hit found	N/A	N/A	N/A	No hit found	N/A	N/A	N/A
Rv3722c	No hit found	N/A	N/A	N/A	No hit found	N/A	N/A	N/A
Rv3031	No hit found	N/A	N/A	N/A	No hit found	N/A	N/A	N/A
Rv2681	No hit found	N/A	N/A	N/A	NP_002676.1	4e-08	25	11
Rv2681	No hit found	N/A	N/A	N/A	NP_001001998.1	5e-08	25	11
Rv0227c	No hit found	N/A	N/A	N/A	No hit found	N/A	N/A	N/A
Rv2613c	NP_215778.1	2e-06	28	12	NP_002003.1	5e-09	32	16
Rv1461	NP_215978.1	1e-17	28	8	No hit found	N/A	N/A	N/A

Of the six proteins classified to be essential as well as functionally linked with cell wall components, (Table 7), no close homologues were observed in human proteome. Literature survey revealed that studies have listed all the genes except Rv0227 to be plausible targets for drug design [86], [84]. Hence this is the first study to report Rv0227c as a novel target for *MtbH37Rv.*

### Conclusions

Comparative genomics of genes involved in cell envelope biosynthesis amongst the 21 mycobacterial species at different levels of biocomplexity *viz*., sequence similarity, metabolic pathway context and phylogenetic profiling provide a rationale for the observed variation in components of cell wall according to their niche occupancy. Our findings suggest that the genes involved in mycolate biosynthesis are highly conserved with variations observed in genes, which form cylcopropane rings. AG biosynthesis is conserved in all the 21 *Mycobacteria*. LM/LAM biosynthetic machinery is conserved with known-variations in capping. PDIM-specific polyketide synthases are present only in pathogenic strains. The predicted functional linkages augment the search space responsible for the biosynthesis of the crucial components of cell wall apart from providing a rationale for the analyzing of network hubs and understanding the subtle relationships between various pathways. Experimental data can further validate the specific function encoded by proteins predicted through phylogenetic profiling studies in different metabolic pathways. Moreover, the shortlisted probable drug targets provide a hypothesis for use in tuberculosis drug design and needs to be tested experimentally. The methodology addresses several issues related to annotation discrepancies amongst closely related organisms apart from providing broader network of genes involved in any metabolic process. The conserved genes complement the TraSH data to arrive at a catalogue of ‘minimal gene set’ that *Mycobacteria* require for their survival and hence pathogenicity. The variant gene-set suggest the existence of alternate routes for biosynthesis of the cell envelope components. The methodology used is robust and is applicable for analyses of hundreds of prokaryotic genomes that are being sequenced due to the advent of NGS technologies.

## Supporting Information

Table S1 Complete list of predicted functionally linked genes involved in mycolate biosynthesis.
(XLS)

Table S2 Complete list of predicted functionally linked genes involved in Arabinogalactan biosynthesis.
(XLS)

Table S3 Complete list of predicted functionally linked genes involved in Lipomannan and lipoarabinomannan biosynthesis.
(XLS)

Table S4 Complete list of predicted functionally linked genes involved in Phthiocerol dimycocerosate biosynthesis.
(XLS)
